# Introducing Serine as Cardiovascular Disease Biomarker Candidate via Pathway Analysis

**DOI:** 10.31661/gmj.v9i0.1696

**Published:** 2020-02-10

**Authors:** Mostafa Rezaei Tavirani, Mona Zamanian Azodi, Mohammad Rostami-Nejad, Hamideh Morravej, Zahra Razzaghi, Farshad Okhovatian, Majid Rezaei-Tavirani

**Affiliations:** ^1^Proteomics Research Center, Faculty of Paramedical Sciences, Shahid Beheshti University of Medical Sciences, Tehran, Iran; ^2^Proteomics Research Center, Shahid Beheshti University of Medical Sciences, Tehran, Iran; ^3^Gastroenterology and Liver Diseases Research Center, Research Institute for Gastroenterology and Liver Diseases, Shahid Beheshti University of Medical Sciences, Tehran, Iran; ^4^Skin Research Center, Shahid Beheshti University of Medical Sciences, Tehran, Iran; ^5^Laser Application in Medical Sciences Research Center, Shahid Beheshti University of Medical Sciences, Tehran, Iran; ^6^Physiotherapy Research Center, Shahid Beheshti University of Medical Sciences, Tehran, Iran

**Keywords:** Metabolome, Metabolic Networks, Cardiovascular Diseases

## Abstract

**Background:**

The rate of death due to cardiovascular disease (CVD) is growing. Investigations about CVD that leading to introduce varieties of metabolites is available. The monitoring of these metabolites to find effective ones in the future of clinic applications is the main aim of this study.

**Materials and Methods:**

Numbers of 34 metabolites for the CVD are extracted from literature and designated for interaction determinations by MetScape V 3.1.3. The compound-reaction-enzyme-gene network was constructed and the pathways were analyzed. Based on the presence of metabolites in the pathways the critical compounds were determined.

**Results:**

Pathway analysis revealed 18 disturbed pathways related to the CVD. glycerophospholipid metabolism pathway including 27 compounds is related to the 9 queried metabolites. L-Serine which was communed between 5 pathways and also was presented in the largest pathway was identified as the critical compound.

**Conclusion:**

It can be concluded that L-Serine is a proper biomarker candidate for CVD diagnosis and also patients follow up approaches.

## Introduction

The rate of death due to cardiovascular disease (CVD) e.g., coronary heart disease, stroke, and rheumatic heart disease is mainly increased. This augmented rate is reported about 22% from 1999 to 2005 [1]. There are estimations about the growing mortality rate of CVD in several regions of the world due to the increment of risk factors such as tobacco consumption, hypertension, physical inactivity, depression, obesity, and diabetes mellitus [2]. Molecular mechanism investigation provides useful information related to diseases that could be used in their management [3]. High throughput methods such as proteomics, genomics, and metabolomics are the advanced method used widely to investigate molecular aspects of many diseases [4-6]. In the metabolomics studies, differentially levels of metabolites such as lipids, amino acids, and organic acids in the patient samples relative to the controls are evaluated [7]. There are several researches in terms of metabolome characteristics via systematic analysis to discover new compounds relative to the diseases [8-10]. Since metabolites level changes study could provide new insight into the molecular profile of CVD, a review article that collected metabolites and discussed in detail about their roles in CVD is selected [11]. Such studies lead to the introduction of large numbers of differential metabolites related to the disease. To reduce the number of these metabolites and achieve to the effective ones, network analysis is an appropriate procedure. In this approach, the metabolites, genes, or proteins are interacted with each other to construct an interactome unit. Analysis of interactome provides informative data that help to screen the queried metabolites [12-14]. On the other hand, pathway analysis is an attractive method to find biochemical pathways that are correlated to the queried metabolites. In this way, metabolites are connected to the relevant pathways that a schema of different types of pathways and distribution of metabolites among them is accessible [15,16]. In the present study, pathway analysis is applied to screen metabolites related to the CVD to find effective ones in clinical practice.

## Materials and Methods

The KEGG ID (https://www.genome.jp/kegg/compound/) of CVD related metabolites from the previous systematic review study in 2017 by Miguel RuizCanela *et al*. [11] were derived. In the mentioned study, terms related to metabolomics and CVD were searched in MEDLINE and EMBASE and also related references. Among 629 downloaded records, number of 12 qualitative articles were selected based on study criteria and then were considered for analysis. More details about data collection are available in the original published review[11]. The list of compounds associated with the CVD were then searched against MetScape V 3.1.3; the Cytoscape application to visualize the network of connections between these compounds and surrounding molecules. The Cytoscape used in our study was the version of 3.7.1 that analyzed these metabolites [17]. MetScape provides information relative to the human metabolome by the query of compounds and genes. Metabolites can either be introduced by the KEGG ID or their name in the query. For genes as well, either name or Entrez Gene IDs works for the software recognition. Here, the KEGG IDS are applied for the query of pathways that these metabolites participate and interact with each other and other molecules. For network constructions, two options are available in MetScape including pathway-based and correlation-based. The network type was set to compound-reaction-enzyme-gene. By applying the pathway-based filter as one of the options of network enriching, the reaction between these metabolites, genes, and enzymes can be visualized and deciphered as pathway networks. This plug-in arranges metabolites data from NCIBI that is combined data of HUMDB, EHMN, and KEGG [18]. The most highlighted pathways with the highest number of metabolites could be valuable in the disease mechanism. In addition, metabolites with the highest number of frequency in the network of compound-reaction-enzyme-gene could suggest more importance.

## Results

**Figure 1:**
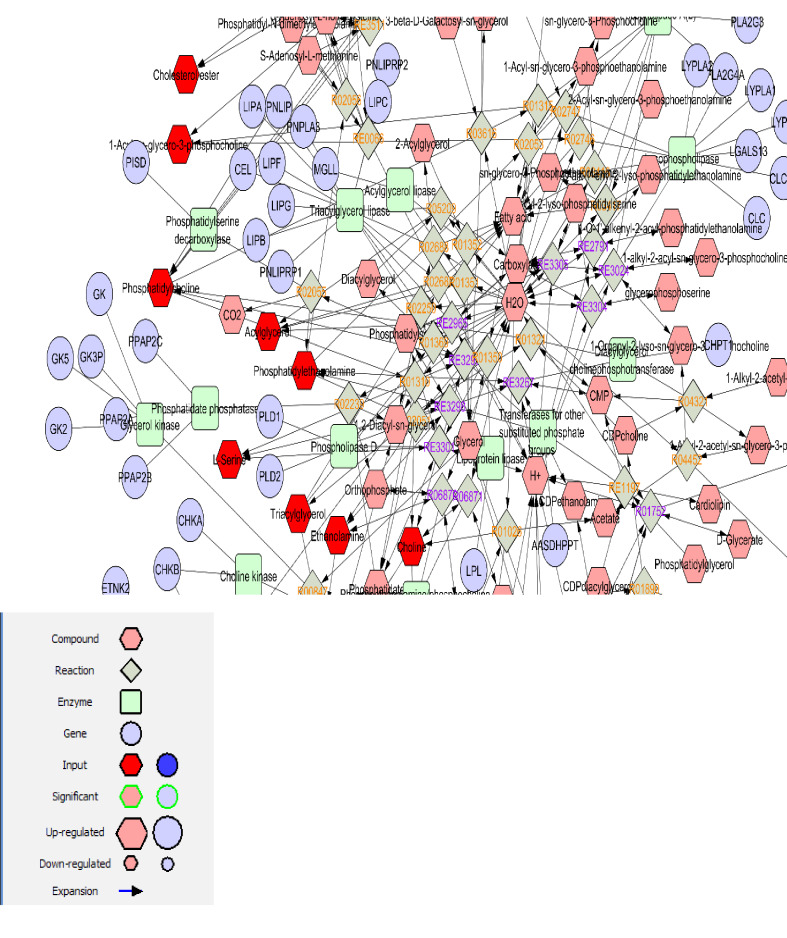
Zoomed part of the glycerophospholipid metabolism pathway. Node colors and shapes indicate different molecules.

A number of 34 compounds linked to cardiovascular disease were identified with KEGG IDs from KEGG Website (Table-1). The function of metabolites can be predicted from the network connections via MetScape an online software, Cytoscape plug-in (http://apps.cytoscape.org/apps/metscap). Except for 3-Methylhistidine (C01152), creatinine (D03600), and TMAO (M00455), the other compounds were found in the MetScape query. A network of compound-reaction-enzyme-genes was retrieved by this application. The obtained network of the query metabolites has 527 nodes and 608 connections. This network is composed of 18 subnetworks (pathways). The list of pathways in this network is tabulated in Table-2. There are seven pathways with more than one metabolites from the queried individuals are gathered (Table-2). Glycerophospholipid metabolism showed the highest amount of both input and additional compounds among other pathways (Figure-1). Glycerophospholipid metabolism is the top-ranked pathway based on compound-reaction-enzyme-gene network analysis. It consists of 27 metabolites that among them 9 were from the inputs. A number of 30 genes contribute to this pathway.

## Discussion

The discovery of CVD related biomarkers could be useful in the diagnosis of disease and also for treatment purposes. In a way, it is possible to reduce the mortality rate and approve the life quality of patients. Metabolite biomarkers have attracted the attention of scientists historically [19]. Lipids and sugars and also their derivatives are well-known biomarkers that are used vastly in clinics [20]. Usage of limited numbers of sensitive and specific biomarkers related to a certain disease in diagnosis (such as glucose in diabetes) is established from many years ago. Advances in biomarker discovery methods provided new opportunities to introduce efficient biomarkers or set of biomarkers (a panel) associated with the studied diseases [21]. Proteomics, genomics, metabolomics and other high throughput methods are used widely to identify new biomarkers. In this regard, it is feasible to obtain large numbers of differential proteins, genes, or metabolites that are correlated to the investigated disease. As it is shown in Table-1, the number of 34 differentially metabolites are presented in relationship to CVD. An important step in the analysis of these metabolites for selecting the suitable ones is screening action. Numbers of nine amino acids and three amino acid derivatives (about 35% of all introduced metabolites) are listed in Table-1. Lipids and organic acids are the second large group that are highlighted. It seems that determination of effective one is a difficult procedure. This difficulty is seen about the amino acid metabolites; which ones are proper biomarkers? To solve this problem; network analysis is a useful method that is common in a wide range of investigations. In Table-2, 18 different pathways linked to the queried metabolites are listed. As it is shown in this table, the introduced pathways are characterized by numbers of nodes, connections, and the presence of numbers of queried metabolites. Considering the name of the listed pathways, important biochemical pathways are presented such as tricarboxylic acid cycle, glycerophospholipid metabolism, bile acid biosynthesis, urea cycle and metabolism of arginine, proline, glutamate, aspartate and asparagine, and other important pathways. Therefore, it is impossible to select a certain one as a significant pathway; a pathway that reflects events in the CVD. Since the screening of metabolites and the selection of limited numbers of effective ones is the main aim of this analysis, pathway analysis is a method to introduce critical metabolites. As it is tabulated in Table-2, there are 11 pathways that include one number of the queried metabolites. Among these 11 pathways, eight individuals include the compounds that are common with the other ones (the pathways including at least two queried metabolites). The other three ones; Butanoate metabolism, tricarboxylic acid cycle, and tryptophan metabolism are related to 2 **-**hydroxybutanoic acid, citrate, and tryptophan respectively. On the other hand; the glycerophospholipid metabolism pathway as the largest pathway is related to the nine queried metabolites and has common metabolites with nine other pathways. Details of elements and connections in this pathway are presented in Figure-1. It can be concluded that dysregulation of this pathway will affect the half numbers of the total pathways. Thus, the glycerophospholipid metabolism pathway can be considered as a sensitive pathway that is connected with the CVD. Some input compounds such as L-Serine are highly contributed to the retrieved pathways. L-Serine is connected to the five pathways and it is presented in the glycerophospholipid metabolism pathway. It seems that this amino acid is a suitable candidate in correlation with CVD. Based on an investigation in 2019 [22]; amino acid catabolism and biosynthesis, fatty acid oxidation, interferon-gamma, and cellular defense response are differentially changed in the hypertensive nephrosclerosis patients. In this document alteration in urine execration of 11 amino acid including serine is reported [22]. Distribution of amino acid metabolism counting serine, fatty acid oxidation, and tricarboxylic acid cycle in human nephrosclerosis biopsies via pathway analysis is reported by Liu *et al*. (2018) [23]. Direct blood pressure–lowering effects of serine is reported by Mishra *et al*. [24]; hence, it can be expected that decrement of serine leads to a significant alteration in blood pressure control. This conclusion is confirmed and the highlighted role of serine and dopamine in this regard is discussed [22]. Correlation between serine level and five pathways including the glycerophospholipid metabolism pathway as the largest individual in our analysis and confirmative documents imply that serine is a suitable candidate relative to the CVD. Since biomarker discovery is an attractive approach in the diagnosis and treatment of diseases, it seems that serine is a suitable compound to be evaluated in the blood of adequate numbers of patients in comparison with controls as a biomarker candidate in CVD.

## Conclusion

 Pathway analysis revealed that among 34 reported metabolites, serine could be considered as a potential diagnostic biomarker nominee in CVD patients. It can also be utilized as a biomarker in the follow-up of patients. Yet, more investigation in this regard is suggested for possible application of serine in clinics.

## Conflict of Interest

 The authors declare no conflict of interest.

## Acknowledgment

 Shahid Beheshti University of Medical Sciences supports this research with the grant number of 15477.

**Table 1:** The List of 34 Metabolite Names Related to Cardiovascular Disease and Their KEGG IDs

**Row**	**Compound Name**	**KEGG ID**
**1**	Carnitine	C00487
**2**	Cholesteryl Ester	C02530
**3**	Triacylglycerol	C00422
**4**	Phosphatidylcholine	C00157
**5**	Choline	C00114
**6**	2-Hydroxybutyrate	C05984
**7**	Betaine	C00719
**8**	Alanine	CE0469
**9**	Citrate	C00158
**10**	Ethanolamine	C00189
**11**	Glucose	C00293
**12**	Glutamate	C00302
**13**	Leucine	C00123
**14**	Lysoalkylhosphatidylcholine	CE6221
**15**	Monoglyceride	C01885
**16**	L-Isoleucine	C00407
**17**	D-Ornithine	C00515
**18**	phenylalanine	C00355
**19**	hydroxyproline	C01157
**20**	5-Oxoproline	C01879
**21**	Serine	C00065
**22**	Sphingomyelin	C00550
**23**	ethanolamine	C00350
**24**	Tryptophan	C00078
**25**	L-Valine	C00183
**26**	Acetylcarnitine	C02571
**27**	Citrulline	C00327
**28**	TMAO	M00455
**29**	creatinine	D03600
**30**	Lysophosphatidylcholine	C04230
**31**	3-Methylhistidine	C01152
**32**	tyrosine	C00082
**33**	2-Hydroxybutyrate	C05984
**34**	Docosahexaenoic acid	CE0328

**Table 2:** The List of Pathways Derived from the Compound-Reaction-Enzyme-Gene Network and Their Associated Network Properties.

**Pathway name**	**NN**	**NL**	**NC**	**NQM**	**QM**
**Glycerophospholipid metabolism **	126	158	27	9	1-Acyl-sn-glycero-3-phosphocholine^*^,Acylglycerol, Cholesterol ester^**^, Choline, Ethanolamine^***^, L-Serine^, Phosphatidylcholine^^^^, Phosphatidylethanolamine^^^^^, Triacylglycerol
**Urea cycle and metabolism of arginine, proline, glutamate, aspartate, and asparagine **	55	55	17	5	5-Oxoproline, Carnitine^*^^, L-Citrulline, O-Acetylcarnitine, trans-4-Hydroxy-L-proline
**Glycine, serine, alanine and threonine metabolism **	44	42	15	4	Betaine, Choline, L-Serine^, gama-L-glutamyl-L-alanine
**Valine, leucine and isoleucine degradation**	34	34	13	3	L-Isoleucine, L-Leucine, L-Valine
**Tyrosine metabolism **	86	102	15	2	3,4-Dihydroxy-L-phenylalanine, L-Tyrosine^**^^
**Glycosphingolipid metabolism**	19	18	5	2	L-Serine^, Sphingomyelin
**Linoleate metabolism**	22	21	3	2	1-Acyl-sn-glycero-3-phosphocholine^*^ , Phosphatidylcholine^^^^
**Tryptophan metabolism**	25	24	8	1	Tryptophan
**Tricarboxylic acid cycle**	21	25	7	1	Citrate
**Methionine and cysteine metabolism**	14	16	6	1	L serine^^^
**Phosphatidylinositol phosphate metabolism**	22	21	4	1	Phosphatidylethanolamine^^^^^
**Biopterin metabolism **	7	6	4	1	L-Tyrosine^**^^
**Vitamin B9 (folate) metabolism**	8	7	4	1	L-Serine^^^
**Lysine metabolism**	7	7	3	1	Carnitine^*^^
**Prostaglandin formation from arachidonate**	7	6	3	1	Ethanolamine***
**Butanoate metabolism **	9	8	2	1	2-Hydroxybutanoic acid
**Arachidonic acid metabolism **	21	20	2	1	Phosphatidylcholine^^^^
**Bile acid biosynthesis **	10	10		1	Cholesterol ester^**^

**NN:** Number of nodes; **NL:** Number of links; **NC:** Number of compounds; **NQM:** Number of queried metabolites; **QM:** Queried metabolites Common compounds are represented by a similar combination of the two characters; * and ^.
